# Artificial Intelligence for Identifying the Prevention of Medication Incidents Causing Serious or Moderate Harm: An Analysis Using Incident Reporters’ Views

**DOI:** 10.3390/ijerph18179206

**Published:** 2021-08-31

**Authors:** Marja Härkänen, Kaisa Haatainen, Katri Vehviläinen-Julkunen, Merja Miettinen

**Affiliations:** 1 Department of Nursing Science/Faculty of Health Sciences, University of Eastern Finland, 70211 Kuopio, Finland; kaisa.haatainen@kuh.fi (K.H.); katri.vehvilainenjulkunen@uef.fi (K.V.-J.); 2 Kuopio University Hospital, 70210 Kuopio, Finland; merja.miettinen@kuh.fi

**Keywords:** artificial intelligence, hospital, incident report, medication incident, prevention

## Abstract

The purpose of this study was to describe incident reporters’ views identified by artificial intelligence concerning the prevention of medication incidents that were assessed, causing serious or moderate harm to patients. The information identified the most important risk management areas in these medication incidents. This was a retrospective record review using medication-related incident reports from one university hospital in Finland between January 2017 and December 2019 (n = 3496). Of these, incidents that caused serious or moderate harm to patients (n = 137) were analysed using artificial intelligence. Artificial intelligence classified reporters’ views on preventing incidents under the following main categories: (1) treatment, (2) working, (3) practices, and (4) setting and multiple sub-categories. The following risk management areas were identified: (1) verification, documentation and up-to-date drug doses, drug lists and other medication information, (2) carefulness and accuracy in managing medications, (3) ensuring the flow of information and communication regarding medication information and safeguarding continuity of patient care, (4) availability, update and compliance with instructions and guidelines, (5) multi-professional cooperation, and (6) adequate human resources, competence and suitable workload. Artificial intelligence was found to be useful and effective to classifying text-based data, such as the free text of incident reports.

## 1. Introduction

Medication intake can treat ailments and prolong the life of patients but can also cause serious harm as medication errors are extremely common [1]. Based on the definition by the National Coordinating Council for Medication Error Reporting and Prevention (NCC MERP), a medication error (ME) is: ‘any preventable event that may cause or lead to inappropriate medication use or patient harm while the medication is in the control of the health care professional, patient, or consumer’ [2]. In this study, we concentrate only on MEs made and reported by health care professionals.

The majority of MEs do not cause serious harm to patients, but some are serious and can even cause death [3]. MEs are a leading cause of harm in health care globally, with an annual estimated cost of 42 billion USD, at least one death per day and injuries to 1.3 million people annually in the USA. Thus, the World Health Organization has prioritised medication safety as a global patient safety challenge [4].

MEs can occur at any stage during the medication process. Since most MEs are preventable, effective medication safety processes are needed [5]. However, MEs are difficult to reduce. In complex health care systems, MEs result from an interplay of multiple factors. These contributing factors to errors are, for example, related to health professionals (such as accuracy, following the guidelines, responsibility, and attitude, skills), teams (such as flow of information and division of work) and organisations (such as work environment, resources, training), [6], as well as factors related to patients and medications.

Worldwide, health care organisations gather information on incidents into incident reporting systems to identify areas for improvement. Incident reports include both structured and unstructured (free text) information. Free text information is valuable for identifying contextual factors that may contribute to such incidents [7]. However, manual analysis of the free texts can be challenging using traditional qualitative text-based analysis methods, as datasets are usually large [8]. Thus, thorough and timely human review of incident reports is challenging owing to its volume and velocity [9]. Hence, data science solutions through automation might be beneficial for solving problems related to the volume and velocity of data.

Artificial intelligence (AI) has been described as the ‘science and engineering of making machines, especially intelligent computer programs’ [10]. The system by which computers can mimic human cognitive functions is called an AI system [11]. Data mining, ontologies and semantic reasoning, clinical decision support systems, smart homes, and medical big data are some areas covered by AI [12]. AI techniques fall into two major categories. First, machine learning (ML) techniques analyse structured data, such as imaging, genetic and electrophysiological data [13]. The second category includes natural language processing (NLP), which extracts information from unstructured data, such as clinical notes or incident reports. NLP targets the change of unstructured texts into machine-readable structured data, which can then be analysed using ML techniques [14].

Several studies have confirmed the high effectiveness of NLP in extracting structured information from unstructured free texts [8,15,16]. Within data science, NLP is a domain which attempts to understand, process, and interpret human language. NLP uses computational techniques to structure these unstructured data, and consequently, these structured data, provide a basis for machine learning models to analyse incident reports more automatically reducing the human workload and the analysis time [17]. Still, NLP and text mining, as well as qualitative analysis methods require significant input from the researcher during the analysis process. Thus, in this this study we used a novel AI based programme called Aiwo that can analyse the textual data inputted to systems fully automatically.

As factors contributing to MEs are manifold and complex, including multiple confounders, there is no single possibility to correct these problems. We need a more detailed understanding of health professionals’ views on how these could and should be prevented, and especially information on how to prevent incidents causing moderate or serious harm to patients. For this purpose, we will analyse incident reporters’ views using AI to guide this analysis. The specific aim is to describe incident reporters’ views identified by AI concerning the prevention of medication incidents causing serious or moderate harm to patients. The information will identify the most important risk management areas of these medication incidents. In addition, this study demonstrated the usability of automatised AI program for analysing this type of free text datasets. To the best of our knowledge, this is the first time that this type of analysis has been conducted.

## 2. Materials and Methods

### 2.1. Design and Setting

This was a retrospective record review that used AI. Medication-related incident reports from a university hospital in Finland were used for this analysis.

### 2.2. Data

Medication-related incidents that were reported between the 1 January 2017 and 31 December 2019 at a university hospital were used. These incidents were anonymously and voluntarily reported using the hospital’s web-based incident reporting system called HaiPro. HaiPro is used in over 200 social service and health care organisations in Finland to learn from such incidents [18]. A total of 3496 medication-related incidents were reported during this period in this hospital. The severity of incidents (degree of harm) was evaluated by handlers of incidents afterwards (Table 1).

### 2.3. Data Analysis

We used the Aiwo system (developed by Aiwo Digital Ltd., Jyväskylä, Finland) real-time qualitative analytics program that combined AI technologies and qualitative research processes. The Aiwo system service does not require separate vocabulary training when the data are brought to the service. Therefore, the Aiwo system’s qualitative content analysis is fully data-driven and does not include any keywords. With this system, it is possible to understand phenomena, trends, changes, and relations between different themes and topics [19].

The Aiwo system is based on NLP, text mining (including clustering and concept linking) and qualitative research, which is guided by AI by fully automatically classifying data at different levels. Original authentic descriptions verified the identified categories. The system is based on algorithms that can be taught automatically through machine learning and enabling them to classify the material. In unsupervised learning, machine learning algorithms tend to group or cluster cases into different categories.

HaiPro incident report data from the hospital was sent using a secure e-mail in excel format to Aiwo Oy. All incident reports were inputted to the Aiwo system by the Aiwo personnel. We used the following filters in the Aiwo system for collecting specific incidents under analysis: (1) medication-related incidents, (2) incidents classified as causing serious or moderate harm to patients, and (3) only information concerning incident reporters’ views about preventing such incidents. In this study, moderate harm was defined (based on the HaiPro, Finnish incident reporting system classification) as causing additional suffering, injury causing some treatment or action, prolonged care, or delays in treatment having health effects. Serious harm was defined as injuries that significantly reduce patients’ quality of life, require life-sustaining treatment, or cause disability or death.

The Aiwo system made the analysis fully automatic based on the selected filters and categorised the data into four main categories, sub-categories, and themes within these categories. Direct quotes using authentic incident reports confirmed these themes and categories. Based on these findings, the most important areas for serious and moderate medication incidents risk management were identified by agreement of all authors.

### 2.4. Ethics

Research permission was obtained from the hospital in the spring of 2021. We used anonymous incident reports, thus the guarantee the anonymity of the reporters, patients, other involved persons, and organisations. Ethical approval was not required according to the National Ethics Committee [20], because the research was based solely on registry and documentary data. The Finnish National Board on Research Integrity’s ethical principles of research were followed, and all data handling was conducted following the ‘responsible conduct of research’. Based on this, the research was conducted so that it did not cause significant risks, damage or harm to research participants and researchers respected the dignity of research participants, including the right to privacy [20]. Due to nature of incident report data, it was not possible to make it openly available.

## 3. Results

**Figure 1:**
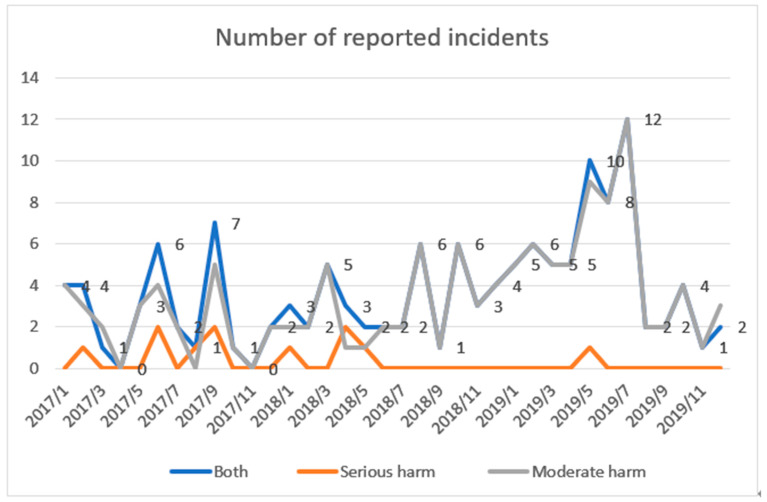
Number of reported incidents causing serious or moderate harm (n = 137) years 2017–2019.

The dataset included medication-related incidents reported between 1 January 2017 and 31 December 2019, of which 11 and 126 cases were assessed as causing serious and moderate harm, respectively (Figure 1, Table 1). These incidents (n = 137) were selected for the analysis.

### 3.1. Prevention of Serious Medication Incidents

The Aiwo system classified the main categories according to the following: (1) treatment, (2) working, (3) practices, and (4) setting. The most common subcategories are listed in Table 2 and described as follows:

#### 3.1.1. Treatment

Under this category, the most common sub-categories were: ‘Drugs’, ‘Medication’, ‘Infusions and hydration’, ‘Operations, and ‘List of medicines’. The most common shared themes with other categories and examples are shown in Table 3.

#### 3.1.2. Working

Under this category, the most common sub-categories were: ‘Carefulness’, ‘Nurses’, ‘Physicians’, ‘Time schedules’, and ‘Changes’. The most common shared themes with other categories and examples are shown in Table 4.

#### 3.1.3. Practices

Under this category the most common sub-categories were: ‘Guidelines’, ‘Prescriptions and recommendations’, ‘Documenting’, ‘Data management and protection’, and ‘Flow of information and communication’. The most common shared themes with other categories and examples are shown in Table 5.

#### 3.1.4. Setting

Under this category, the most common sub-categories were: ‘Recovery room’, ‘Machines’, and ‘Data processing systems: Miranda, Oberon, and Pegasos ja Clinisoft’. The most common shared themes with other categories and examples are shown in Table 6.

### 3.2. Most Important Areas for Risk Management of Medication Incidents

Based on the categorization of reporters’ views on preventing medication incidents that caused serious or moderate harm, the following risk management areas were identified:

(1) Verification, documentation and up-to-date drug doses, drug lists and other medication information;
(2) Carefulness and accuracy in managing medications;
(3) Ensuring the flow of information and communication regarding medication information and safeguarding continuity of patient care;
(4) Availability, updations, and compliance with instructions and guidelines;
(5) Multi-professional cooperation;
(6) Adequate human resources, competence, and suitable workload.

## 4. Discussion

This study described incident reporters’ views concerning the prevention of medication incidents assessed causing serious or moderate harm to patients by using AI for data analysis. The AI programme called Aiwo made the analysis fully automatic, which is a novel approach. Thus, this study also demonstrated the usability of an automatised AI program for analysing these types of free text datasets. These kind of automatised methods are important as the amount of data in health care, including number of incident reports, is constantly growing and effective analysis methods are thus required. These methods are especially required for analysing text-based datasets, such as free-text descriptions of incident reports that incude valuable views of the reporters. This study demonstrated that automatised methods can produce valid and useful results for improving clinical practice and significantly saving analysis time and manpower.

The information produced by the Aiwo system classification identified the most important areas for risk management of serious medication incidents. The findings were similar to those found in previous studies. For example, a manual qualitative analysis of medication administration related incident reports by Härkänen et al. [6] found the following categories related to health professionals: (1) accuracy and preciseness, (2) verification and (3) following the guidelines, responsibility and attitude toward work, teams: (1) distribution of work, (2) flow of information and cooperation and (3) documenting and marking the drug information and organisations: (1) work environment, (2) resources, (3) training, (4) guidelines, and (5) development of the work. Thus, our analysis indicated that AI could classify incidents meaningfully since the sub-categories were similar in both studies, even though the main categories were different in the manual and AI analysis. The difference between these main categories (manual: made by a human and AI analysis: made by the Aiwo system) are explained by different datasets as well as by the interpretation of researcher (human and AI in this case) that is required in qualitative based analysis. Thus, variability between interpretations and different researchers in naming or classifying categories is understandable.

Previous studies have identified many ways to prevent medication incidents. Computerised prescriber order entry (CPOE) systems have been generalised in health care, but studies have described contradictory findings regarding effectiveness in ME prevention. Some studies have shown a significant reduction in non-intercepted serious MEs [21], while others found that CPOE is expensive to install and update [22] and has no effect on administration errors [23]. Our study findings were also related to data processing systems, documentation, flow of information, data management and protection, and patient data, demonstrating that even though this kind of technology exists, these are not always used as effectively and carefully as required. Additionally, the guidelines and division of work between health professionals remain unclear, which may cause the information to be recorded or informed incorrectly resulting in MEs.

Unclear medication information, doses, and omitted verifications practices were found in our study, even though the study hospital partly used superior technology, such as automated dispensing cabinets (ADCs). In a previous study conducted in the same hospital, ACDs were found to make work easier, but some resistance to change was observed in the form of non-compliance to some instructions, for example, the barcode was not always used [24]. Following the guidelines and verification practices are important in ME prevention, but as found in earlier studies, more than half of the nurses often fail to follow guidelines during medication administration in Finland [25] and rule violations are common globally [26]; these are not only nursing problems. The root causes for these rule violations and guideline nonadherence should be clarified in the future to improve patient safety.

The parenteral drug administration process is vital for administering intravenous medications in critically ill in-patient management; however, errors associated with this route of MA are extremely common and can be equally serious to the patients [27]. In our studies, these themes were found in the categories describing infusions and machines. The use of smart infusion pumps can improve medication safety to some extent. However, to achieve the most medication safety, institutional support and behavioural improvement of nursing personnel is required along with well-designed technological tools [28]. Most of these are due to skill and knowledge deficiencies. Some errors are also routine violations, which are learned workplace behaviours [27].

Liang and Gong [29] also used AI for the classification of patient safety reports, which is considered as the primitive step in performing further analysis. The study focussed on the importance of understanding the multi-labelled nature of patient safety reports to understand the course and development of medical errors.

### Strengths and Limitations

Our study has several strengths as well as some limitations. Incident reporting has been an ongoing practice in the study hospital for 14 years. Health professionals are used to reporting such incidents. However, possible underreporting [30], and reporting bias could affect the number, type, and temporality of reported incidents and data interpretation [31,32]. In addition, reported severity ratings are only indicative evaluations and some inconsistencies in severity ratings may be caused by a lack of understanding of how to report the ‘degree of harm’ [33]. Thus, this may introduce some risk of bias related to selected data, but it was not possible to verify this. Still, evaluations of severity ratings are made afterwards by the experienced incident data handlers, not reporters, as it is not possible to know the real severity at the time the event occurs.

One limitation is the data size including fewer than 200 incidents. The reason for this is that severe and moderate incidents are rare. This might affect generalisation of these results, as well as the use of only one hospital record. Thus, the results are only indicative, but support further research in this field using AI software.

We had a close collaboration with AI experts holding special language and cultural anthropology expertise, thereby increasing the study validity. The AI application with NLP combined with data from the incident reporting system makes these databases efficient and offers many benefits, including its effectiveness. Previous studies have also discussed the effectiveness of this kind of analysis. For example, text mining and machine learning are effective in reducing the human workload by extracting the necessary information [34]. Similarly, a study that evaluated the time-saving nature of NLP systems compared to manual review found that for every 1 h of NLP system development, there was a time savings of 20 h of manual review [35]. Still, these kinds of AI systems are kind of ‘black-box’ methods, researchers in this study did not know exactly what the algorithms behind the system were. This study demonstrated the clinical usefulness of the system, but in the future, more attention should be drawn to methods to ensure the reproducibility of these kind of automatised analysis.

## 5. Conclusions

Health systems and professionals should make every effort to ensure careful medication management, by following guidelines and verifications, and with skilled professionals’ effective communication and cooperation. These areas should be targeted in safety improvement interventions and continuing education in health care. In addition, health care leaders should guarantee that modern technology, resources, and organisations’ safety culture support medication safety improvement. AI was found to be useful and effective for classifying text-based data, such as the free text of incident reports. This kind of rapid analysis can be useful for producing important information for improving clinical practice in real time. In addition, it produced much more useful information about necessary development measures that would have been possible by using only structured information of incident reporting system.

## Figures and Tables

**Table 1:** Severity of medication-related incidents (n = 3496) reported 2017–2019.

Type of Harm	%	Frequency
No harm	49.8%	1742
Mild harm	18.4%	642
Moderate harm *	3.6 %	126
Serious harm *	0.3%	11
Not known	27.9%	975
Total	100	3496

* used in analysis.

**Table 2:** Main categories and most common sub-categories of preventing serious and moderate medication incidents organised by artificial intelligence.

**Treatment:** - Drugs - Medication - Infusions and hydration - Operations - List of medicines	**Working:** - Carefulness - Nurses - Physicians - Time schedules - Changes
**Practices:** - Guidelines - Prescriptions and recommendations - Documenting - Data management and protection - Flow of information and communication	**Setting:** - Recovery room - Machines - Data processing systems: “Miranda, Oberon, Pegasos ja Clinisoft” - Stocks and cabinets

**Table 3:** Sub-categories and themes under main category “Treatment”.

Sub-Categories	Most Common Shared Themes with Other Categories	Example of the Free Text-Description of Incident (Date of the Incident)
Drugs (n = 39)	- Working: Physicians (n = 11) - Practices: Guidelines (n = 7) - Working: Carefulness (n = 7) - Practices: Prescriptions and recommendations (n = 7) - Working: Nurses (n = 6) - Treatment: Medication (n = 5) - Working: Changes (n = 4) - Treatment: Operations (n = 3) - Practices: Documenting (n = 3) - Working: Emergency (n = 3)	‘Any drug, especially for opioids, should be carried out by careful verification of the strengths of the drug with such care whenever the drug is administered that such a serious drug error would not occur. The drug’s dosage package has a really clearly expressed strength of the drug, so it has been entirely about the serious thought error of the midwife who gave the drug.’ (12 September 2017)
Medication (n = 11)	- Treatment: Drugs (n = 5) - Working: Physicians (n = 2) - Working: Carefulness (n = 2) - Treatment: List of medicines (n = 2) - Practices: Prescriptions and recommendations (n = 2) - Practices: Documenting (n = 2)	‘The physician should record the medications they prescribe, so the correctness of the order would be ensured... At the same time, the doctor would also notice allergies and interactions.’ (5 July 2019)
Infusions and hydration (n = 5)	- Practices: Flow of information and communication (n = 2) - Treatment: Drugs (n = 2) - Treatment: Operations (n = 1) - Treatments: Anaesthesia (n = 1) - Practices: Meetings (n = 1) - Practices: Trainings (n = 1) - Practices: Data management and protection (n = 1) - Setting: Recovery room (n = 1) - Setting: Machines (n = 1) - Working: Time schedules (n = 1) - Setting: Outpatient clinic (n = 1) - Working: Nurses (n = 1)	‘In the middle of the rush, attention to the patient is important and check the infusion pathways that the drug really goes where it should be.’ (14 December 2019)
Operations (n = 5)	- Treatment: Drugs (n = 3) - Treatment: Anaesthesia (n = 2) - Working: Nurses (n = 2) - Practices: Trainings (n = 1) - Practices: Flow of information and communication (n = 1) - Working: Physicians (n = 1) - Setting: Recovery room (n = 1) - Working: Emergency (n = 1) - Treatment: Infusions and hydration (n = 1)	‘A checklist for a patient going into surgery should be submitted to the ward.’ (8 May 2019)
List of medicines (n = 4)	- Working: Carefulness (n = 3) - Treatment: Medication (n = 2) - Working: Physicians (n = 1) - Working: Nurses (n = 1) - Practices: Documenting (n = 1) - Practices: Data management and protection (n = 1)	‘Timely and carefully reviewing the medical list by physician. Nurse should ask if there are all medications at home.’ (1 September 2018)

**Table 4:** Sub-categories and themes under main category “Working”.

Sub-Categories	Most Common Shared Themes with Other Categories	Example of the Free Text-Description of Incident (Date of the Incident)
Carefulness (n = 21)	- Treatment: Drugs (n = 7) - Working: Nurses (n = 4) - Practices: Guidelines (n = 4) - Working: Time schedules (n = 4) - Treatment: List of medicines (n = 3) - Setting: Stocks and cabinets (n = 2) - Working: Physicians (n = 2) - Treatment: Medication (n = 2) - Practices: Documenting (n = 2)	‘Attention and carefulness in the implementation of prescriptions.’ (24 August 2018)
Nurses (n = 16)	- Treatment: Drugs (n = 6) - Working: Carefulness (n = 4) - Working: Physicians (n = 3) - Setting: Recovery room (n = 2) - Working: Changes (n = 2) - Treatment: Doses (n = 2) - Treatment: Operations (n = 2) - Practices: Trainings (n = 2) - Practices: Flow of information and communication (n = 2)	‘Could the incident have been prevented by working as a couple, in which case the inexperienced would “still” have a more ruined nurse with whom to go through daily routines throughout the day and thus ensure that all the work is done.’ (24 July 2019)
Physicians (n = 12)	- Treatment: Drugs (n = 11) - Practices: Prescriptions and recommendations (n = 5) - Practices: Guidelines (n = 5) - Working: Nurses (n = 3) - Working: Carefulness (n = 2) - Treatment: Medication (n = 2)	‘Up-to-date administration entries. In the absence of medications, the physician should be informed if there is no replacement’ (2 October 2017)
Time schedules (n = 5)	- Working: Carefulness (n = 4) - Working: Nurses (n = 1) - Working: Babies and children (n = 1) - Treatment: Drugs (n = 1) - Treatment: Medication (n = 1) - Treatment: Infusions and hydration (n = 1) - Practices: Guidelines (n = 1)	‘Accuracy for calculating infusion rate, unhurried machine programming.’ (27 June 2019)
Changes (n = 4)	- Treatment: Drugs (n = 4) - Working: Nurses (n = 2) - Working: Physicians (n = 1) - Practices: Flow of information and communication (n = 1) - Practices: Prescriptions and recommendations (n = 1)	‘The drug changes were made on Monday afternoon and they were left unnoticed. Changes were left unnoticed over several shifts.’ (7 August 2019)

**Table 5:** Sub-categories and themes under main category “Practices”.

Sub-Categories	Most Common Shared Themes with Other Categories	Example of the Free Text-Description of Incident (Date of the Incident)
Guidelines (n = 12)	- Treatment: Drugs (n = 7) - Working: Physicians (n = 5) - Working: Carefulness (n = 4) - Practices: Documenting (n = 3) - Treatment: Doses (n = 3) - Setting: Stocks and cabinets (n = 2) - Practices: Prescriptions and recommendations (n = 2)	‘Equipment should be familiar and there should be clear guidelines for new equipment.’ (16 January 2018)
Prescriptions and recommendations (n = 9)	- Treatment: Drugs (n = 7) - Working: Physicians (n = 5) - Practices: Guidelines (n = 2) - Treatment: Medication (n = 2)	‘Regular review of the prescriptions section and responding to them. Especially if the on-call physician has been contacted.’ (26 October 2019)
Documenting (n = 5)	- Treatment: Drugs (n = 3) - Practices: Guidelines (n = 3) - Treatment: Medication (n = 2) - Working: Carefulness (n = 2)	‘Carefulness in recording home medication especially when it comes with a clear list of home medications. The patient is not always able to tell when to take any medicine.’ (6 July 2019)
Data management and protection (n = 5)	- Treatment: Drugs (n = 2) - Treatment: List of medicines (n = 1) - Treatment: Medication (n = 1) - Treatment: Infusions and hydration (n = 1) - Practices: Documenting (n = 1) - Practices: Practices and policies (n = 1) - Practices: Meetings (n = 1) - Practices: Guidelines (n = 1) - Practices: Orientation (n = 1) - Practices: Patient record (n = 1) - Practices: Flow of information and communication (n = 1) - Working: Carefulness (n = 1) - Setting: Outpatient clinic (n = 1)	‘There needs to be a mark of how chemotherapy is dripping, how much medicine is left. Now you can’t find information about this even half night, how much fluid went the previous day.’ (8 January 2019)
Flow of information and communication (n = 4)	- Working: Nurses (n = 2) - Treatment: Drugs (n = 2) - Treatment: Infusions and hydration (n = 2) - Treatment: Operations (n = 1) - Treatment: Anaesthesia (n = 1) - Practices: Meetings (n = 1) - Practices: Training (n = 1) - Practices: Data management and protection (n = 1) - Practices: Prescriptions and recommendations (n = 1) - Setting: Recovery room (n = 1) - Working: Carefulness (n = 1) - Setting: Outpatient clinic (n = 1) - Working: Physicians (n = 1) - Working: Changes (n = 1)	‘Staff should ask from patient themselves and check from risk information to see if any previous reactions are known in their medical papers.’ (21 June 2017)

**Table 6:** Sub-categories and themes under main category “Setting”.

Sub-Categories	Most Common Shared Themes with Other Categories	Example of the Free Text-Description of Incident (Date of the Incident)
Recovery room (n = 4)	- Working: Nurses (n = 2) - Working: Babies and children (n = 1) - Treatment: Operations (n = 1) - Treatment: Anaesthesia (n = 1) - Treatment: Medication (n = 1) - Treatment: Infusions and hydration (n = 1) - Practices: Trainings (n = 1) - Practices: Orientation (n = 1) - Practices: Flow of information and communication (n = 1)	‘Maybe there were too many events at the same time. Simultaneous caring of mother and child in the recovery room is hectic, especially after emergency dissection when the patient does not have epidural anaesthesia. In addition, two students were involved. There should be calmness at work and the ability to focus on just one patient.’ (22 February 2017)
Machines (n = 2)	- Treatment: Doses (n = 1) - Treatment: Drugs (n = 1) - Treatment: Infusions and hydration (n = 1) - Practices: Documenting (n = 1) - Practices: Guidelines (n = 1) - Practices: Letters (n = 1)	‘Distrust of newer Space infusion machines was aroused. It’s hard to put a hose on a spiral if it was suspected as a reason. I think the hose was right on the machine.’ (27 March 2019)
Data processing systems: (Miranda, Oberon, Pegasos ja Clinisoft) (n = 2)	- Treatment: Drugs (n = 2) - Working: Physicians (n = 1) - Treatment: Preparations (n = 1) - Practices: Prescriptions and recommendations (n = 1)	‘It would be recommended that the doctor document prescriptions in Miranda [data processing system], hand-transfer of entries expose to error events.’ (17 July 2019)

## Data Availability

The research data is confidential incident reports that should not be shared to any third parties.
